# Mental Health in Women Victims of Gender Violence: Descriptive and Multivariate Analysis of Neuropsychological Functions and Depressive Symptomatology

**DOI:** 10.3390/ijerph19010346

**Published:** 2021-12-29

**Authors:** Ana Victoria Torres García, María Concepción Vega-Hernández, Concha Antón Rubio, Miguel Pérez-Fernández

**Affiliations:** 1 Department of Personality, Psychological Evaluation and Treatment, Faculty of Psychology, Ciudad Jardín Campus, University of Salamanca, 37005 Salamanca, Spain; mipefe@usal.es; 2 Department of Statistics, Higher Polytechnic School of Zamora, Viriato Campus, University of Salamanca, 49029 Zamora, Spain; mvegahdz@usal.es; 3 Department of Social Psychology and Anthropology, Faculty of Psychology, Ciudad Jardín Campus, University of Salamanca, 37005 Salamanca, Spain

**Keywords:** abused women, neuropsychological sequelae, attention, memory, depressive symptomatology, Luria-DNA battery

## Abstract

Female victims of abuse, as well as suffering from psychopathological disorders such as depression, can have neuropsychological sequelae affecting memory and attention, with serious consequences, both physical and psychological, in their daily lives. Therefore, the objective of this study is to analyse these sequelae that affect attention and memory, as well as the possible association of these sequelae to depression. A total of 68 women, victims of gender-based violence, between the ages of 15 and 62 participated in this study. The Luria DNA Battery (Neuropsychological Diagnosis of Adults) by Manga and Ramos (2000); and the Beck Depression Inventory (2011) were applied. It is shown that female victims of gender-based violence present poor short-term memory, attentional control, and score low on the Luria-DNA battery. Of these women, 60% suffer from some relevant type of depression. Through HJ-Biplot analysis, a direct relationship was found between memory and attentional control with the total score of the Luria battery. However, an inverse relationship was found between short-term memory and depression. In addition, three well-differentiated clusters of female victims of gender-based violence were identified. It is concluded that a lower rate of depression is observed in female victims of abuse when they have a more intact short-term memory.

## 1. Introduction

Gender violence is a social scourge that affects women all over the world, and is considered the most brutal example of inequality in our society. In the Spanish legal system, article 1 of Organic Law 1/2004 on Comprehensive Protection against Gender Violence defines it as: “Any act of violence (...) that, as a manifestation of discrimination, the situation of inequality and the power relations of men over women, is exercised against women by those who are or have been their spouses or by those who are or have been linked to them by similar relationships of affection, even without cohabitation. (...) that results in or is likely to result in physical, sexual or psychological harm or suffering to women, as well as threats of such acts, coercion or arbitrary deprivation of liberty, whether occurring in public or private life.” [1].

Gender-based violence includes, among other manifestations, physical, sexual, and psychological violence between people of different genders in an intimate relationship, regardless of their marital status, sexual orientation, or cohabitation [2,3].

Physical violence involves, among other behaviors, punches, kicks, attempted strangulation, blows using any object, etc., as a manifestation of physical abuse [4]. Sexual violence involves behaviors related to the sexual sphere that are perceived as degrading and unwanted by the victim. These forms of violence involve psychological violence against the victim. Psychological violence is the most common and, at the same time, the most difficult to detect due to the different ways in which it manifests itself and will be present simultaneously with the other forms of violence [5]. Among the behaviors of psychological violence that constitute mistreatment are isolation, intimidation, use of threats, cognitively confusing the victim, emotional abuse, economic subjugation, use of minors, and harassment, among others [6,7]. In relation to neuropsychology and gender violence, one of the problems we encounter is that women victims of gender violence, as Marin Torices et al. (2016) point out, are hardly ever evaluated neuropsychologically [8]. These women may present neuropsychological deficits due to, among other circumstances, the blows received in the head by the abuser. In this respect, neuropsychology can provide answers to the symptoms that may be present in these women, related to cognitive processes, or in other words, a neuropsychological evaluation would provide objective information on their neuropsychological state. In our research, we consider the question of whether female victims of violence present some kind of neuropsychological deficit.

The neuropsychological assessment provides a valid description of the strengths and weaknesses of the cognitive profile, in order to be able to plan a specialized treatment tailored to this profile [9,10], without forgetting the affective and personality sphere.

This assessment should be comprehensive, the starting point for neuropsychological rehabilitation. In this respect, the Luria-DNA battery is comprehensive and, at the same time, selective [11]. It is comprehensive because it encompasses the five areas of cognitive functioning, which are considered dominant in the exploration of possible impairment from the perspective of clinical practice. Clinical neuropsychology emerged as a specialty with the aim of identifying, measuring, and describing behavioral changes associated with certain brain dysfunctions. The changes that occur in higher functions have to be identified, measured, and described in certain areas. These abilities include the following areas: visuospatial, language, memory, intellectual processes, and attention [12,13,14]. On the other hand, the Luria-DNA battery is selective, so that it makes it possible to explore the higher psychological processes in a short period of time and meets the criteria of being sensitive to offer a neuropsychological profile with multiple possibilities of comparison, being its strengths and weaknesses interpretable from Luria’s neuropsychological theory [11].

Cognitive deficits are also present in patients with major depression and/or depressive symptomatology, presenting alterations in executive functions [15,16,17], neuropsychological deficits affecting attentional functions [18], information processing speed and motor functioning [19] and episodic memory [15,20]. Alterations in working memory [21] and executive functions [17], are frequent, and they frequently present alterations in verbal fluency tests [22], attentional set shifting [21,23], planning [17,24] and inhibition of automatic responses [21,24].

On the other hand, in cases of childhood anxiety and depression [25], there is impairment in memory functions and cognitive flexibility [26,27], lower performance in attention processes, and attentional control [28,29] in subjects evaluated with depressive symptoms. There is significant evidence showing the presence of alterations, poor functioning, or low neurocognitive performance in attention, memory, and/or executive function in children and adolescents with symptoms or disorders related to anxiety and/or depression [30,31,32,33].

As a consequence of gender violence, women victims of this type of violence may present neuropsychological sequelae that will affect their lives and especially their daily lives [34], since they present a diversity of physical, psychological, neurological, and cognitive problems [35,36,37]. This population group is a particularly vulnerable group that requires not only psychological, but also neuropsychological attention [38].

Currently, there is no doubt that this type of violence affects the quality of life and well-being of the victims. These consequences may appear immediately, but on other occasions, they manifest later on and may become irreversible as a consequence of the cumulative effect of violent episodes [39,40,41,42,43]. It is considered, in this study, whether women victims of gender violence scored low in the total score of the Luria-DNA battery and the areas of memory and attention tests.

Neuropsychological research related to gender violence is still scarce, although the results indicate that there are important neuropsychological sequelae [1,34,44]. Research refers, among others, to attentional and memory problems, deficits in executive functions, and processing speed [45].

Abused women, when compared to non-abused women, perform worse in tasks of alternating attention, short-term memory, and direct and indirect visual memory [46], these results are in the same line as those found by Twamley et al. [47]. The areas of memory, attention, and concentration are especially compromised in that women show greater distractibility problems, significant difficulties in concentration and in remembering everyday events, as well as in contained and sustained attention as a consequence of having been exposed to violence. Other areas that affect the visuoconstructive ability and executive functions, as well as fluency speed and motor processing, are also impacted, affecting decision-making and response inhibition [37,47,48,49,50,51].

On the other hand, research on gender-based violence and autobiographical memory is still unexplored, despite the fact that the impact of autobiographical memory dysfunction in everyday life has already been demonstrated. Billoux et al. indicate that there is autobiographical memory dysfunction in women victims of gender-based violence, and follows a pattern similar to this type of memory deterioration in victims of traumatic events [52].

According to Torres and Pérez-Fernández women who have suffered gender-based violence score lower in the execution of the different areas of the Luria DNA neuropsychological battery when compared with a group of non-abused women [51]. This shows that there is a neuropsychological impact in the visuospatial area (visual perception and spatial orientation), language area (receptive speech and expressive speech), memory area (short-term memory and logical thinking), intellectual area (thematic drawings and conceptual activity), and in the attention test (attentional control). These results are in line with other studies that show how women victims of abuse show cognitive problems related to physical and psychological abuse, as well as the chronic stress produced by it [49,53].

The cognitive sequelae of gender-based violence due to the direct damage as a result of blows received to the head, as well as indirect damage from exposure to violence that causes psychopathologies, such as post-traumatic stress disorder (PTSD), which entails cognitive and psychological sequelae [54]. On the other hand, injuries caused by blows to the head produce problems in attention and executive functioning [1]. Other studies in this line show that violence is related to a greater neuropsychological deficit and important psychopathological symptoms. Poor results in working memory would be related to high levels of stress, and there are significant differences that relate abuse to impairment in long-term and visual working memory, although no significant differences were found regarding psychopathological symptoms, such as anxiety, depression, stress, and cognitive deficits [46]. In our research, we asked ourselves whether women victims of abuse present depressive symptomatology.

Experiencing victimization may negatively affect women’s emotional stability. In addition to physical injuries caused by violence, they may present depression, anxiety, post-traumatic stress disorder, suicidal ideation, somatizations, low self-esteem, disability and death, and behavioral problems related to substance use and suicide attempts [55,56].

Some authors consider that this may be due to the independent analysis of the relationship between psychological maltreatment and psychopathological symptoms and, on the other hand, physical maltreatment, and cognitive deficits [46].

In our research, we will analyze the association of neuropsychological sequelae related to memory, attention, and depressive symptomatology.

## 2. Materials and Methods

### 2.1. Participants

The study sample was made up of 68 women victims of gender-based violence. 40.63% suffered this type of violence for more than 10 years, 18.75% for less than 3 years, and 40.63% between 4 and 10 years. The participants’ age ranged between 15 and 62 years and lived in Spain at the time of the data collection.

### 2.2. Procedure

In order to obtain the sample, due to the peculiarities and access difficulties (especially for protection reasons), a collaboration of public and private organizations that work with this population was required (Law enforcement forces: Civil Guard, Corps National Police Corps, Local Police, and Autonomous Police; Social Action Centers (CEAS in Spanish), associations and shelters for women victims of violence, hospitals, and universities, among others). The study was presented by the research team to the different institutions involved. First, a personal interview was arranged with the head of each institution in order to inform them of the characteristics of the research, as well provide them with brochures and information cards that could facilitate contact with women victims of violence who could be referred to participate in the study. As such, the selection of the participants was carried out through consecutive sampling, selecting women who met the inclusion criteria specified in the protocol as they were recruited. These criteria are: Being between 16 and 65 years of age at the time of the examination, a criterion that was necessary to be able to apply the Luria-DNA battery; having an IQ between 90 and 100 or above, which corresponds to an average score or above; no addictions to any type of drugs that could interfere with the test results; being or having been a victim of gender violence in any of its manifestations at the time of being evaluated; the violence could be exercised by any person who had an effective relationship with the victim (partner, parent, sibling, child, etc.); being in the process of deciding to leave the abusive situation or to have left it; not presenting a diagnosis of specific pathology related to the abuse, depression, anxiety, PTSD, etc. although they could present certain comorbid symptoms with these pathologies.

### 2.3. Ethical Aspects

The participants signed a consent document at the time of conducting the interviews. This protocol was approved by the research ethics committee of the Institute of Women and the Ministry of Labor and Social Affairs. Project identification code: 6T0116. Date of approval: 2003. Declaration of Helsinki.

### 2.4. Instruments

Luria-DNA battery (Neuropsychological Diagnostic of Adults). The Luria-DNA battery [11] is a neuropsychological assessment and diagnosis instrument for adults based on the model created by Luria. This battery completes neuropsychological evaluations in all evolutionary stages, as it can be applied to subjects between 16 and 65 years of age. The Luria-DNA battery meets the criteria of thoroughness and time required in all neuropsychological batteries, according to Kolb and Whishaw [57], and is defined as comprehensive and selective at the same time [11]. It is comprehensive because it encompasses all the five areas of cognitive functioning that are considered dominant in the exploration of a possible deterioration from the perspective of clinical and selective practice. This means it allows the exploration of higher psychological processes (visuospatial area, language, memory, intellectual processes, and attention), in a short period of time (approximately 40 min of the test when performed by trained professionals). The battery contains 81 items, distributed in eight subtests.

Among the areas explored by this instrument is memory, one of the most important fields of neuropsychological exploration, with two subtests that evaluate two different types of memory: Short-term memory and Logical thinking.

Short-term memory: Evaluates a type of memory linked to the perception process and includes the consolidation of impressions of the subject. It explores the learning process through five trials of a series of unrelated words that must be remembered in any order. Additionally, it explores retention and evocation in verbal and non-verbal tasks. Verbal memory is examined in a broader way using words, numbers, and phrases, sometimes with the presence of interferences. This exploration is completed with the presentation of a story in which the subject has to indicate its essential semantic components, which serves at the same time as interference of certain verbal orders.

Logical thinking: Evaluates a type of memory linked to complex intellectual processes. The deficit in the use of active auxiliary aids at the service of mnesic and intellectual processes is associated with frontal lobe dysfunctions. Indirect memorization is explored through relationship processes that are established between words and cards, or between expressions and drawings made by the subject itself. In addition, attention plays a fundamental role in cognitive functioning, thus, it is another of the key areas that the Luria-DNA battery evaluates.

Attentional control: Aims to find out the capacity for attention control or functioning. Attention-concentration is analyzed using opposite verbal and non-verbal responses, where the subject has to select responses that conflict with common ones, requiring the inhibition of easier and automated responses. Response association, their omission, or sounds that are difficult to discriminate from each other are also explored. Lastly, sustained attention is explored in following words that do not contain a specific vowel sound. With these items, the momentary state of the selective and sustained attention capacity of the evaluated subject is tested.

Beck Depression Inventory. The Beck Depression Inventory is one of the most widely used self-report instruments to assess depressive symptoms in adolescents and adults [58,59].

The BDI consists of 21 items to assess the intensity of depression. In each of the items, the subject must choose the statement from a set of four alternatives, always in order of severity, that best approximates his or her average state during the last week including the day on which he or she completes the inventory. Each item is rated, according to the severity scale, from 0 to 3 points, depending on the alternative chosen. The total score of the 21 items ranges from 0 to 63. In the event that the subject chooses more than one alternative in a given item, only the score of the most severe statement chosen is considered. Finally, Weight Loss (item 19) is only assessed if the subject indicates that he/she is not on a weight loss diet. If he/she is, a score of 0 is given for the item.

A diagnosis is made based on criteria or cut-off scores that define different categories or levels of severity of depressive symptomatology based on the total score obtained from each individual. The cut-off scores and degrees of depression are as follows: 0–13 indicates minimal depression, 14–19 mild depression, 20–28 moderate depression, and 29–63 severe depression.

### 2.5. Data Analysis

A descriptive analysis of the variables related to the neuropsychological diagnosis and depressive symptomatology was carried out to assess cognitive functioning and possible sequelae in women victims of gender-based violence. The instructions of the Beck depression inventory’s authors were followed to categorize the women according to depressive symptomatology.

To analyze possible relationships between the variables under study, a bivariate correlation analysis was carried out and a regression analysis was performed afterwards. The latter consisted of two analyses: a model with the total score of the Luria-DNA battery as the explanatory variable, and a second model taking into account the neuropsychological variables. Furthermore, in order to analyze in greater depth, the relationships between the variables in abused women victims, a multivariate analysis was performed using HJ-Biplot by Galindo-Villardón [60]. This biplot method allows women, depression, and neuropsychological variables to be represented in a single graph of the highest quality of representation, which means that their relationships can be interpreted visually. It uses vectors as points called markers j_1_, j_2_,…, j_n_ for each row, and vectors called markers h_1_, h_2_, …, h_p_ for each column. Each row represents a woman and each column a variable, such that both markers’ sets can be superimposed onto the same reference system. This method is based on the singular value decomposition of the multivariate data matrix **X_nxp_**: **X = U D V’** where **J = U D** and **H = D V’**. **U** is the matrix whose columns are the eigenvectors of **XX^T^**, **V** is the matrix whose columns are the eigenvectors of **X^T^X**, and **D** is the matrix diagonal of singular values λ_i_ of **X**. For the interpretation, it must be taken into account that the variables are represented as vectors and the individuals (women in this case) as points in the graph, the distance between the points indicates the dissimilarity between women victims of gender-based violence, the length of a vector expresses variability of the variable, the cosine of the angle between vectors indicates a correlation between variables, and the projection of a point on a vector indicates the relationship between the woman and the variable. In addition, HJ-Biplot provides information in terms of inertia, so that the more variability, the more information, thus, the more inertia. The measure of the relationship between the biplot’s axes of representation and each of the observed variables is the so-called relative contribution of factor to element and represents the part of the variability of each of the variables explained by factor. This contribution will allow us to know which are the variables most directly related to each axis and, therefore, the variables responsible for the placement of the individuals on projections on each of the axes.

Lastly, the biplot coordinates were analyzed using a hierarchical cluster analysis.

The data were analyzed using the IBM SPSS Statistics package [61] and the biplotbootGUI R package [62].

## 3. Results

### 3.1. Neuropsychological Functions and Depressiove Symptomatology in Women Victims of Gender Violence

The results of the Luria-DNA battery in women victims of gender-based violence indicate a low total score (M = 38.13, SD = 18.07). Paradoxically, these women have the highest scores in expressive and receptive speech (M = 48.97, SD = 11.15 and M = 46.03, SD = 11.08), followed by conceptual activity (M = 42.84, SD = 10.23). The average score obtained in short-term memory, spatial orientation, attentional control, and thematic drawings is around 40 points (M = 41.32, SD = 14.98; M = 40.96; SD =13.83; M = 40.74, SD = 18.31; M = 40.07, SD = 15.16; respectively). As expected, the lowest score corresponds to visual perception and logical thinking (M = 39.93, SD = 16.17 and M = 37.21, SD = 19.44).

The mean score in the Beck depression inventory for women who have experienced gender-based violence is 18.36, with a standard deviation of 11.95. 59.02% of women victims of gender-based violence were classified with some relevant type of depression: 14.75% with mild depression, 24.59% with moderate depression, and 19.67% with severe depression, while 40.98% of the women were classified with minimal depression.

### 3.2. Association between Neuropsychological Sequelae and Depressive Symptomatology

Table 1 presents the Pearson correlation coefficients between the subscales scores of the Luria-DNA battery, as well as the total Luria-DNA score and the Beck depression inventory. We observe direct and highly significant linear relationships between the scores of visual perception, spatial orientation, receptive speech, expressive speech, short-term memory, logical thinking, thematic drawings, conceptual activity, attentional control, and the Luria-DNA total score, on the other hand, these neuropsychological variables appear inversely related to Beck’s total depression score. Significant correlations were found with receptive speech (*p* = 0.030), expressive speech (*p* = 0.043), logical thinking (*p* = 0.024), thematic drawings (*p* = 0.015) and the total score of the Luria-DNA battery (*p* = 0.010), but above all, a highly significant relationship with spatial orientation and short-term memory (*p* = 0.008 and *p* < 0.001). This indicates that as scores on the oral language, memory, spatial orientation, thematic drawings variables, or the Luria-DNA battery increase, scores on the depression inventory decrease. Furthermore, we did not observe significant relationships between depressive symptomatology and visual perception, conceptual activity, or attentional control.

We explored the effects of neuropsychological evaluation on the depressive symptomatology of women victims of gender-based violence. To this end, we carried out several regression analyses in which the Beck depression inventory score variable was regressed to the total score of the Luria-DNA battery, and subsequently to the neuropsychological variables (Spatial orientation, Receptive speech, Expressive speech, Short-term memory, Logical thinking, and Thematic drawings).

The results of the first model (Table 2) show that the total score of the neuropsychological evaluation could explain 10.8% of the variance of depressive symptomatology in women victims of abuse. The estimated value of B_1_ indicates that the value of the score in depression decreases by −0.211 for each point increase in the total score of the Luria-DNA battery. However, the results of the second model show an increase of 1.4% in the variance of depression. These results show that women who have suffered gender-based violence and scored high in short-term memory have lower depressive symptomatology values. The variables of spatial orientation, oral language, logical thinking, and thematic drawings were not significant, and only the short-term memory coefficient was found significant (*p* < 0.05) for model fit.

The HJ-Biplot allows a detailed examination of the variables for the neuropsychological and depressive symptomatology evaluation of women victims of abuse through a multivariate graphic representation.

The percentage of inertia explained by the first three axes is 70.51%, with the first axis being the one with the greatest explained variability (53.71%), the second explaining 9.55%, and the third 7.24%.

Table 3 shows that neuropsychological variables contribute substantially to the factorial axis 1, but are low to axis 2 and 3. The total score of the Luria-DNA battery, expressive speech and short-term memory contribute fundamentally to factor axis 1, while receptive speech, and logical thinking, in addition to contributing to this axis, also do substantially to axis 3. And visual perception and attentional control do so severely to axis 2. The depression variable contributes mainly to axis 2.

**Figure 1:**
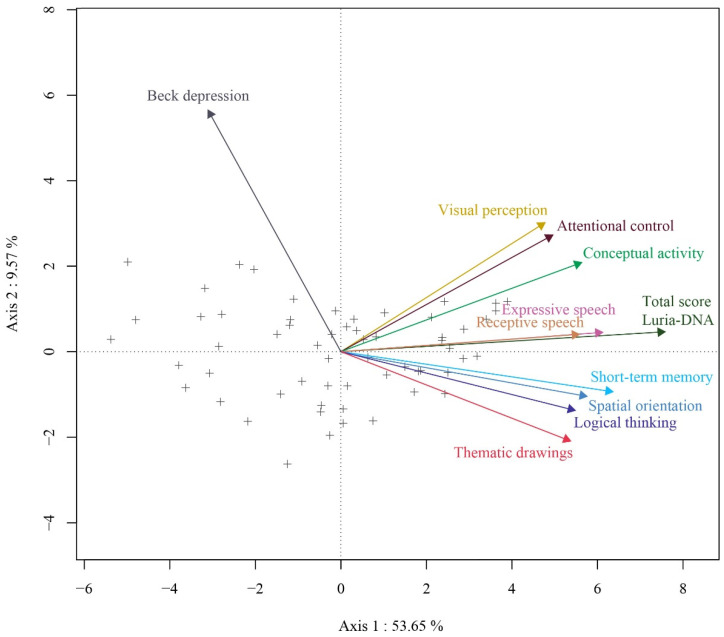
2D graph of HJ-Biplot factorial axis 1–2.

Figure 1 shows the graphic representation of the HJ-Biplot in the 1–2 plane, where it clearly shows the direct relationship between visuospatial, language, memory, intellectual processes, and attention variables with the total score of the Luria-DNA battery, as the angles formed by the vectors of these variables are acute. The high correlation between expressive and receptive speech and the total score of the Luria-DNA battery is also noteworthy (forming an angle whose opening is close to 0 degrees). In addition, the association between visual perception, attentional control, and conceptual activity on the one hand, and the relationship between short-term memory, spatial orientation, logical thinking, and drawings on the other, is noteworthy. This last group of variables appears inversely related to depressive symptomatology, forming an angle close to 180 degrees. The independence of visual perception, attention control, and conceptual activity with depressive symptomatology in women victims of gender-based violence is also shown.

All the variables have noticeable variability due to the length of the vectors, where the vector corresponds to depressive symptomatology being the longest.

Taking into account that women located in similar positions in the graph present a similar behavior and that the plot of each woman with respect to a variable reflects the response of a woman on the said variable, there are women who have suffered gender-based violence with high scores in the neuropsychological evaluation variables, and low in depressive symptomatology, or vice versa.

**Figure 2:**
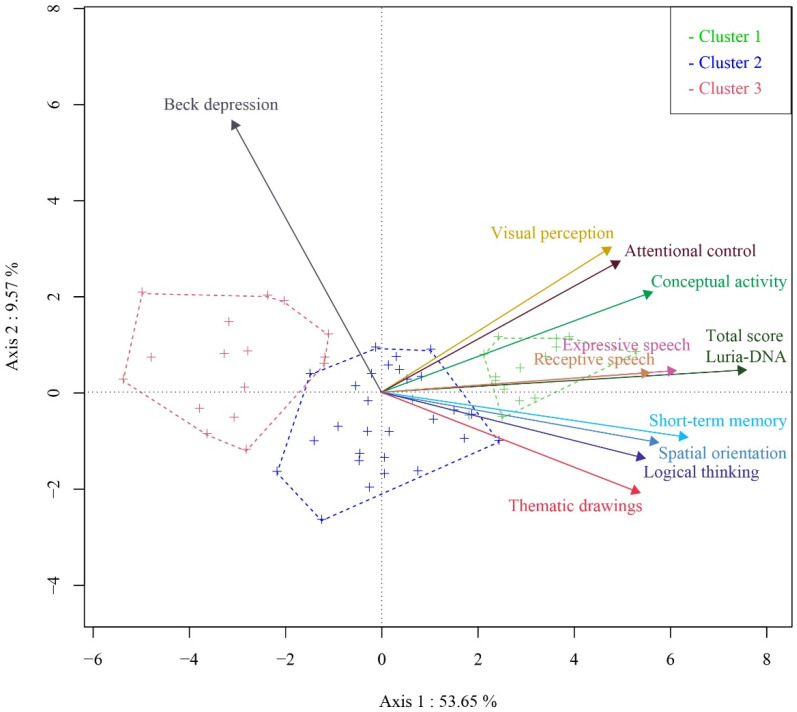
2D graph of HJ-Biplot factorial axis 1–2 by Clusters.

We distinguish three groups of women victims of gender-based violence (Figure 2):

- Cluster 1 consists of 23.3% of women in the analyzed sample who have high scores in all subscales scores of the Luria-DNA battery (with average scores above 50); and low scores in depressive symptomatology (M = 16.86, SD = 5.26).
- Cluster 2 consists of 50.0% of women in the sample who have scores around 40 points in the subscales scores of the Luria-DNA battery; and very low scores in depressive symptomatology (M = 12.93, SD = 7.97).
- Cluster 3 consists of 26.7% of women in the sample who have very low scores in all subscales scores of the Luria-DNA battery; and high scores in depressive symptomatology (M = 28.88, SD = 15.13).

## 4. Discussion

The results of this study show that women victims of gender-based violence who score low on the Luria-DNA battery suffer neuropsychological sequelae. As other research has already shown, abuse can affect brain functioning, and these neuropsychological deficits are related to serious cognitive difficulties related to visuoconstructive skills, motor processing speed, fluency, and executive function, which affect the daily functioning of women and influence memory, attention, concentration, and decision-making [35,36,37,63,64].

Neuropsychological evaluation’s results of the Luria-DNA battery indicate in battered women, higher scores in the area of language and conceptual activity, a high mean score in short-term memory and attentional control, and a low score in visual perception and logical thinking, results in line with other research on attentional problems, memory, and processing speed [46]. Most of the women in our sample have low short-term memory and attentional control, as well as a low total score of the Luria-DNA battery in the same way that has been shown in other research on performance in attentional and memory tasks [46,47].

More than half of the women victims of gender-based violence presented some type of depressive symptomatology. These results are consistent with those obtained by [65,66]. We found significant differences in depressive symptomatology between the groups of women victims of gender-based violence, which relate to the level of short-term memory and the total score of the Luria-DNA battery. This suggests a relationship between short-term memory and depressive symptomatology, coinciding with researchers such as Saeedi et al. who attempted to diagnose depressive disorder from short-term memory [66].

In regards to the relationship that could exist between neuropsychological evaluation and the depressive symptomatology of women victims of gender-based violence, we found that women who have suffered gender-based violence and obtain higher scores in short-term memory have lower scores in depressive symptomatology. These results do not differ from those obtained in other studies, which found that maltreatment is related to psychopathological symptoms, a greater number of neuropsychological deficits, and that working memory is affected by high levels of stress [46].

Through the HJ-Biplot we found a direct relationship between the memory-related variables and attentional control with the total score of the Luria-DNA battery, highlighting a high correlation between logical thinking and the total score of the battery. On the other hand, there is an inverse relationship between short-term memory and depressive symptomatology. If gender violence is related to the duration of the abuse, it is observed that 66.67% of the victims who suffered for three years showed symptoms of depression. In those who endured it for more than 10 years, 70.00% also showed depressive symptomatology. However, in female victims of gender violence abused for between 4 and 10 years, 58.33% did not show symptoms related to depression.

It can be observed that of the women who showed some very mild depressive symptoms, 58.33% suffered abuse for between 4 and 10 years while 25.00% endured it for more than 10 years and 16.67% for less than three years. Moreover, among women with mild depressive symptomatology, 50.00% suffered abuse for between 4 and 10 years, although among those with moderate or severe depressive symptomatology, the majority suffered abuse for more than 10 years (44.44% and 66.67%, respectively).

Research carried out on the duration of abuse indicates that it is possible that certain personality traits may facilitate its endurance [67]. Contrary to what we might expect, getting used to this situation no longer protects women from possible psychological consequences, as they have low levels of self-esteem, difficulties in the ability to adapt, or psychological discomfort, which interferes with partner interactions. This limits the meaningful activities that can be done together [68]. Social interaction is also particularly compromised, as the isolation to which they are subjected have repercussions that lead to deficits in social skills, assertiveness, and the capacity for initiative and decision-making. The insecurity they experience facilitates a greater degree of conformity and submission, as well as feelings of guilt [69], negative thoughts about themselves, and low self-esteem increase the risk of depression [70] as well as greater difficulty in adapting to daily life and a greater risk of suicidal behaviors [71,72]. Despite living in a situation of violence, some women perceive positive aspects of the relationship as expressions of affection and cohesion with their partner, which makes it more difficult to abandon the abuser [73]. The psychological cost of remaining in an abusive context is so high that it can only be borne, to a certain extent, if cognitive distortions and inappropriate coping strategies are present [74].

Our work also has some limitations. One is that this is a cross-sectional study that in future research could also be considered longitudinally. The difficulty of access to the sample of women victims of violence is reflected in the different age groups of the sample. As these are clinical cases, there is a range limitation and, despite the sample size and that the groups do not differ significantly in important demographic variables (age, socioeconomic level, employment status, ...), it would be required to expand the sample size in order to obtain more conclusive results.

Despite these limitations, this study has shown the neuropsychological consequences of attention and memory-related to depressive symptomatology in women victims of gender-based violence. This line of research continues to be novel in terms of the few studies carried out in the area of neuropsychology and gender violence. Research in this field can help psychology professionals to understand more accurately the neuropsychological affectation and the difficulties of women in decision-making, as well as a correct diagnosis for a better response to psychotherapy. Additionally, they can also be useful to other disciplines such as legal, being neuropsychological evaluation a tool in judicial processes, especially in the difficulty to demonstrate certain types of violence, such as psychological. In the area of social services, it would facilitate the victim’s access to community resources, requiring special support and sensitivity from professionals to address this problem. In addition, adequate work is essential so that they can rebuild their lives with their children with the best-guaranteed protection.

## 5. Conclusions

Women victims of gender violence present low neuropsychological performance. They show impairment in the memory area, obtain lower scores in the Luria DNA battery in logical thinking, visual perception and thematic drawings, followed by attentional control, with the highest scores obtained in speech and short-term memory.

Neuropsychological impairment in attentional control could explain part of the errors that women victims of gender-based violence make in their daily lives, such as difficulty in remembering daily events, or problems with attention or concentration.

A high percentage of women victims of gender-based violence presented some type of depressive symptomatology. The low scores obtained by abused women in the area of memory and in the attention test, as well as the total score obtained in the Luria-DNA battery, could have an association with these symptoms, which are present in depressive disorders, this could explain certain errors and forgetfulness frequently referred to by victims of abuse, although it cannot be affirmed that this is due to a neuropsychological impairment or depressive symptomatology.

From the multivariate analysis, a direct relationship was found between memory and attentional control with the total score of the Luria-DNA battery, and an inverse relationship between short-term memory and depressive symptomatology. In relation to these results, three well-differentiated clusters of women victims of gender-based violence were identified:

- The first cluster comprises of women who score high on all subscales of the Luria-DNA battery and obtain low scores in depressive symptomatology
- The second cluster includes women who obtain intermediate scores in all subscales of the Luria-DNA battery and score very low in depressive symptoms.
- And the third cluster is made up of women in the sample who have very low scores on the subscales of the Luria-DNA battery and score high in depressive symptomatology.

## Figures and Tables

**Table 1:** Correlations between neuropsychological and depressive variables studied.

	Visual Perception	Spatial Orientation	Receptive Speech	Expressive Speech	Short-term Memory	Logical Thinking	Thematic Drawings	Conceptual Activity	Attentional Control	Total Score Luria-DNA	Beck Depression
Visual perception	1	0.462 **	0.446 **	0.428 **	0.466 **	0.355 **	0.278 *	0.466 **	0.387 **	0.601 **	−0.125
Spatial orientation		1	0.383 **	0.452 **	0.552 **	0.542 **	0.551 **	0.480 **	0.361 **	0.711 **	−0.338 **
Receptive speech			1	0.595 **	0.596 **	0.370 **	0.486 **	0.518 **	0.335 **	0.725 **	−0.278 *
Expressive speech				1	0.650 **	0.653 **	0.489 **	0.558 **	0.474 **	0.782 **	−0.260 *
Short-term memory					1	0.517 **	0.494 **	0.525 **	0.448 **	0.816 **	−0.445 **
Logical thinking						1	0.531 **	0.418 **	0.355 **	0.708 **	−0.289 *
Thematic drawings							1	0.448 **	0.354 **	0.702 **	−0.312 *
Conceptual activity								1	0.489 **	0.748 **	−0.177
Attentional control									1	0.640 **	−0.148
Total score Luria-DNA										1	−0.328 *
Beck depression											1

Note: ** *p* < 0.01 * *p* < 0.05.

**Table 2:** Regression analysis for neuropsychological variables predictive of depressive symptomatology.

Model		B	SE_B_	Beta	t	*p*	*R* ^2^	*F*	*p*
1	(Constant)	26.228	3.399		7.717	<0.001	0.108	7.013	0.010
	Total score Luria-DNA	−0.211	0.080	−0.328	−2.648	0.01			
2	(Constant)	31.463	7.512		4.188	<0.001	0.122	2.368	0.042
	Spatial orientation	−0.060	0.143	−0.071	−0.421	0.675			
	Receptive speech	0.001	0.181	0.001	0.005	0.996			
	Expressive speech	0.138	0.210	0.128	0.658	0.513			
	Short-term memory	−0.320	0.144	−0.404	−2.221	0.031			
	Logical thinking	−0.016	0.109	−0.026	−0.147	0.884			
	Thematic drawings	−0.095	0.127	−0.123	−0.745	0.460			

Note: Dependent variable: Total score of the Beck depression.

**Table 3:** Relative contribution of factor to element.

	Axis 1	Axis 2	Axis 3
Visual perception	692.73	276.94	30.33
Spatial orientation	804.98	26.19	168.83
Receptive speech	759.38	4.32	236.31
Expressive speech	993.30	5.27	1.43
Short-term memory	900.87	19.45	79.68
Logical thinking	669.34	42.04	288.62
Thematic drawings	769.56	117.7	112.74
Conceptual activity	871.69	117.13	11.18
Attentional control	766.81	233.16	0.03
Total score Luria-DNA	995.54	3.73	0.73
Beck depression	195.08	645.69	159.24
